# Ferroptosis and multi-organ complications in COVID-19: mechanisms and potential therapies

**DOI:** 10.3389/fgene.2023.1187985

**Published:** 2023-05-26

**Authors:** Qi Li, Zeyuan Chen, Xiaoshi Zhou, Guolin Li, Changji Zhang, Yong Yang

**Affiliations:** ^1^Department of Pharmacy, Sichuan Academy of Medical Sciences & Sichuan Provincial People’s Hospital, School of Medicine, University of Electronic Science and Technology of China, Chengdu, China; ^2^Personalized Drug Therapy Key Laboratory of Sichuan Province, School of Medicine, University of Electronic Science and Technology of China, Chengdu, China; ^3^Department of Pharmacy, Luxian People’s Hospital, Luzhou, China; ^4^ School of Basic Medicine and Clinical Pharmacy, China Pharmaceutical University, Nanjing, China

**Keywords:** COVID-19, ferroptosis, iron, ROS, GPX4, multi-organ complications, inhibitors

## Abstract

COVID-19 is an infectious disease caused by SARS-CoV-2, with respiratory symptoms as primary manifestations. It can progress to severe illness, leading to respiratory failure and multiple organ dysfunction. Recovered patients may experience persistent neurological, respiratory, or cardiovascular symptoms. Mitigating the multi-organ complications of COVID-19 has been highlighted as a crucial part of fighting the epidemic. Ferroptosis is a type of cell death linked to altered iron metabolism, glutathione depletion, glutathione peroxidase 4 (GPX4) inactivation, and increased oxidative stress. Cell death can prevent virus replication, but uncontrolled cell death can also harm the body. COVID-19 patients with multi-organ complications often exhibit factors related to ferroptosis, suggesting a possible connection. Ferroptosis inhibitors can resist SARS-CoV-2 infection from damaging vital organs and potentially reduce COVID-19 complications. In this paper, we outline the molecular mechanisms of ferroptosis and, based on this, discuss multi-organ complications in COVID-19, then explore the potential of ferroptosis inhibitors as a supplementary intervention for COVID-19. This paper will provide a reference for the possible treatment of SARS-CoV-2 infected disease to reduce the severity of COVID-19 and its subsequent impact.

## 1 Introduction

Due to its rapid spread and rising mortality rate, the pandemic brought on by the severe acute respiratory syndrome coronavirus 2 (SARS-CoV-2), also known as Corona Virus Disease 2019 (COVID-19), has had a significant influence on the entire world. Globally, more than 6.8 million fatalities and over 755 million occurrences of COVID-19 have been reported as of February 2023 (Organization, 2023). Some infected individuals exhibit only mild signs and symptoms like fever, tiredness, and a chronic cough, a subset develops severe COVID-19 (Borges do Nascimento et al., 2020). Patients with severe COVID-19, however, may experience immunological and coagulation abnormalities and organ damage to the lungs, heart, kidneys, brain, liver, and other organs (Mehta et al., 2020). In addition, individuals with varying degrees of COVID-19 severity, including those with mild to moderate symptoms, often experience neurological, respiratory, or cardiovascular symptoms that can persist for weeks or months, commonly called “post-COVID-19 syndrome” or “long COVID” (Yong, 2021). Although vaccines for COVID-19 are widely available, the emergence of mutant strains of the virus still poses a threat. While antiviral small-molecule oral drugs such as Paxlovid and Molnupiravir have proven to help avoid hospital stays and death in high-risk COVID-19 patients and have been approved for treatment, they have strict population and timing restrictions to use. There have also been reports of recurrent infection and symptom rebound after a 5-day course of therapy with Paxlovid (Rubin, 2022).

Cell death is a two-edged sword in viral infections (Imre, 2020). It can remove cells infected with SARS-CoV-2, thereby inhibiting virus replication and spread. Still, dysregulated cell death can lead to uncontrolled cellular damage and immune responses, contributing to the multi-organ manifestations observed in COVID-19 patients during acute infection and potentially leading to long COVID. Ferroptosis, a type of cell death brought on by the small molecule erastin, was initially identified in 2012 (Dixon et al., 2012). As opposed to apoptosis, a type of programmed cell death, ferroptosis is primarily brought on by a buildup of intracellular lipid reactive oxygen species (ROS), leading to fatal lipid peroxidation (Hadian and Stockwell, 2020). This process is called ferroptosis because iron ion overload is an essential factor in lipid peroxidation. 4-Hydroxynonenal (4-HNE), a breakdown product of lipid peroxidation leading to ferroptosis, can be used as a marker for ferroptosis. The pathology report of a COVID-19 patient in 2020 showed a decreased lymphocyte count and positive staining of the proximal renal tubules and myocardial tissue, suggesting an association between ferroptosis and organ damage caused by COVID-19 (Jacobs et al., 2020). A growing number of studies now suggest a strong link between ferroptosis and COVID-19. This paper discusses the main molecular mechanisms of ferroptosis and its association with multi-organ complications in COVID-19, providing directions for the potential treatment modalities to weaken the effects of COVID-19.

## 2 Molecular mechanisms of ferroptosis

Production of ROS has been reported to occur in the mitochondrial membrane and the mitochondrial and endoplasmic reticulum membranes (Neitemeier et al., 2017). Mitochondria are the metabolic center in most mammalian cells and are an efficient source of ROS. The imbalance between oxidative and antioxidant systems prevents ROS from being removed. The intrinsic/enzyme-regulated pathways mainly involve the inhibition of glutathione peroxidase 4 (GPX4) (Stockwell et al., 2017). Thus, the primary oxidative and antioxidant mechanisms of ferroptosis are analyzed below.

### 2.1 Oxidation mechanisms

#### 2.1.1 Lipid peroxidation

Long-chain fatty acids, which contain more than two double bonds, are called polyunsaturated fatty acids (PUFA). PUFA is a critical component of cell membranes and is vital in regulating biological functions, including physiological and immune responses (Gill and Valivety, 1997). Due to the large number of double bonds, PUFA has less stability and is highly sensitive to oxygen (Yin et al., 2011). Arachidonic acid (AA) and adrenic acid (AdA) are the lipids most vulnerable to oxidation. Generally, lipid peroxidation can be divided into two types: enzymatic oxidation (with enzymes) and free radical chain reactions (non-enzymatic), through which PUFA can be oxidized and broken down into toxic derivatives such as 4-HNEs and malondialdehyde (MDA) (Feng and Stockwell, 2018).

**FIGURE 1:**
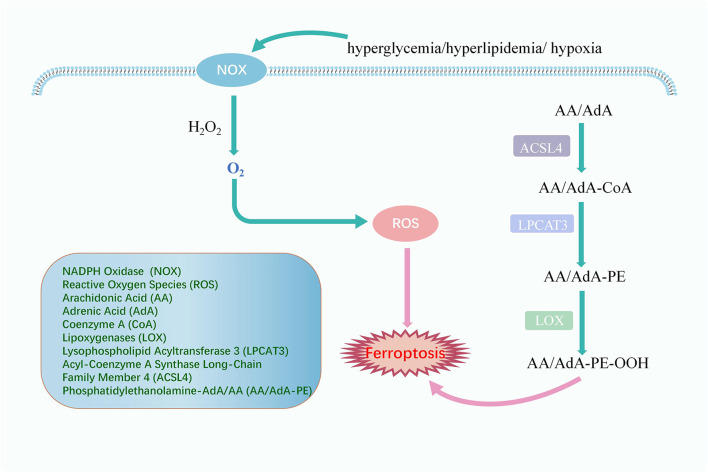
The Signaling Pathway of lipid peroxidation. In hyperglycemia, hyperlipidemia, and hypoxia, NOX catalyzes the reaction of H_2_O_2_ with lipids to produce ROS, leading to ferroptosis. ACSL4 attaches coenzyme A (CoA) to AA/AdA forming AdA-CoA, then LPCAT3 facilitates AdA-CoA esterification in the cell membrane to form AA/AdA-PE which could be directly oxidized to AA/AdA-PE-OOH by LOX, eventually causing ferroptosis.

Several enzyme species are involved in the type of lipid peroxidation caused by enzyme oxidation (Figure 1). The ROS catalyzed by NADPH oxidase (NOX) is the primary source of ROS in the cellular oxidative stress process. Moreover, enzymes such as lipoxygenases (LOXs) are essential in triggering cell ferroptosis (Doll et al., 2019). LOX can directly oxidize phosphatidylethanolamine-adrenic acid/arachidonic acid (AA/AdA-PE) into peroxide products (AA/AdA-PE-OOH), which are ferroptosis signals. However, the conversion of AA and Ada to AA/AdA-PE cannot occur without acyl-coenzyme A synthase long-chain family member 4 (ACSL4) and lysophospholipid acyltransferase 3 (LPCAT3). After ACSL4 ligated coenzyme A (CoA) to AA/AdA, forming AdA-CoA, LPCAT3 facilitates AdA-CoA esterification in the cell membrane to develop AA/AdA-PE (Kagan et al., 2017). The LOX catalytic process contributes to the LOOH cell pool and makes the cells sensitive to ferroptosis. However, some cell lines susceptible to ferroptosis do not express any significant LOX (Shah et al., 2018). Therefore, LOX may not be required in ferroptosis. Non-enzymatic lipid peroxidation is powered by carbon and oxygen-centered free radicals, such as ferroptosis triggered by an iron-dependent free radical mechanism, where it undergoes a free radical chain reaction of lipid peroxidation (Shah et al., 2018).

#### 2.1.2 Iron cycle

**FIGURE 2:**
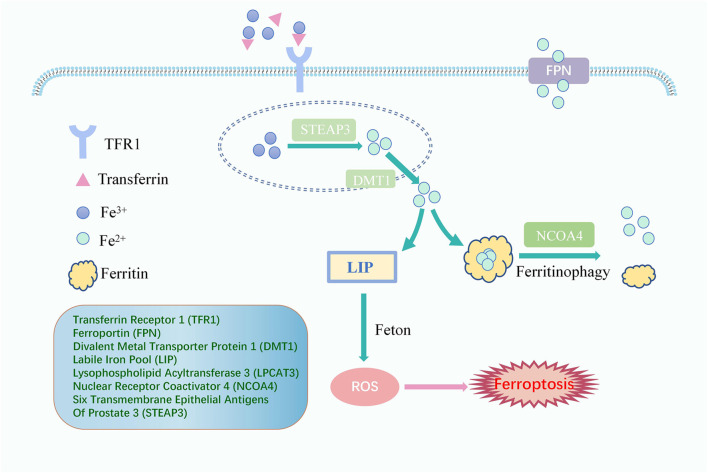
The Signaling Pathway of lipid peroxidation iron cycle. Fe^3+^ binds to transferrin outside the cell and then attaches to TFR1, entering the cell. Fe^3+^ is converted to Fe^2+^ by STEAP3. Fe^2+^ is transported to the cytoplasm via DMT1 to form a LIP which happens Fenton reaction, and excess iron is stored in ferritin. Ferritinophagy, mediated by NCOA4, can degrade ferritin, releasing ferritin-bound iron as free iron, and FPN exports intracellular iron out of cells when needed. Free iron generates ROS through the Fenton reaction, eventually leading to ferroptosis.

Fenton reaction, the oxidation of Fe^2+^ and H_2_O_2_, catalyzes the production of ROS and is the beginning of the non-enzymatic reaction of lipid peroxidation (Hendren et al., 2020). Iron overload is an indispensable link to turning on lipid peroxidation with a critical position in inducing ferroptosis (Figure 2). Usually, iron is absorbed from the intestine into the body as ferrous ions (Fe^2+^) and subsequently oxidized to ferric ions (Fe^3+^). The primary protein for transporting iron is transferrin. Iron ions bind to transferrin outside the cell and then attach to membrane transferrin receptor 1 (TFR1), entering the cell through endocytosis and colonizing the endosome (Andrews and Schmidt, 2007; Frazer and Anderson, 2014). Upon entry into the cell, in acidic endosomes, the six transmembrane epithelial antigens of prostate 3 (STEAP3) decrease Fe^3+^ to Fe^2+^. Fe^2+^ is transported to the cytoplasm via divalent metal transporter protein 1 (DMT1) and eventually liberated into the cytoplasm and mitochondria to form a labile iron pool (LIP). In contrast, excess iron, which forms redox-inactive heterogeneous polymers, is stored in ferritin to protect tissues and cells from damage (Ryu et al., 2017; Philpott, 2018). Ferroportin (FPN), the only known mammalian protein, can export intracellular iron out of cells when needed (Ganz, 2005). Iron import, storage, and export imbalance lead to iron overload (Stockwell et al., 2017).

Worthy of mention is that nuclear receptor coactivator 4 (NCOA4) can promote iron storage or release from ferritin and plays a crucial function in maintaining a dynamic balance of intracellular iron. Indeed, when cellular iron levels are low, ferritinophagy, cell-selective autophagy mediated by NCOA4, can degrade ferritin, leading to the release of ferritin-bound iron as free iron (Santana-Codina and Mancias, 2018). The evidence has shown that NCOA4-mediated degradation of proteins may also be an essential mechanism for the occurrence of ferroptosis. Additionally, Haeggstrom and Funk (2011) introduced the notion of atypical ferroptosis in 2008, demonstrating that an increase in the intracellular LIP caused by iron overload leads to the over-activation of heme oxygenase-1 (HO-1), which ultimately induces atypical ferroptosis.

### 2.2 Antioxidant system

**FIGURE 3:**
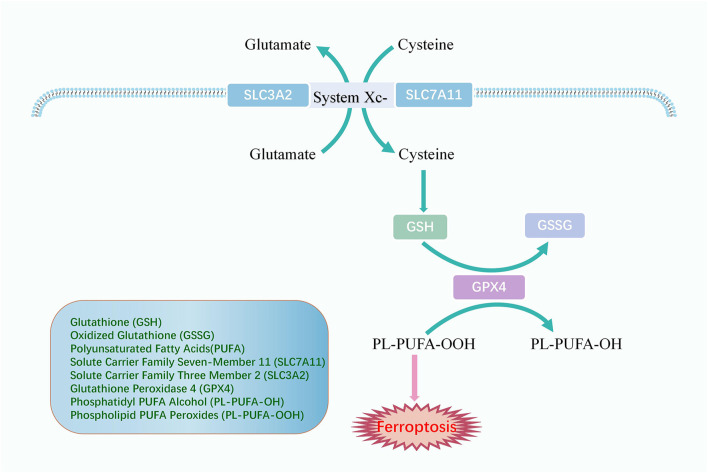
The mechanism of antioxidant system. System Xc-comprises SLC3A2 and SLC7A11 and promotes cellular uptake of cysteine, an essential precursor for GSH synthesis. GPX4 converts GSH to GSSG and returns PL-PUFA-OOH to the PL-PUFA-OH, achieving ferroptosis resistance.

Lipid peroxidation could induce ferroptosis. If the antioxidant is deactivated in the cell, lipid peroxides cannot be removed and thus accumulate, eventually leading to cellular damage and death (Figure 3). Glutathione (GSH) and oxidized glutathione (GSSG) are widely available in cells for controlling intracellular oxidation levels. GSH is mainly available in the cytoplasm and, to a lesser extent, in organelles such as mitochondria and is involved in many biological processes (Dolma et al., 2003). GSH is equipped with the biological function of scavenging ROS to play a crucial role in preventing ferroptosis. Cysteine is an essential precursor of GSH. The solute carrier family seven-member 11 (SLC7A11) and the solute carrier family three member 2 (SLC3A2) comprise the heterodimer referred to as system Xc-, a cysteine and glutamate reverse system (Chen et al., 2021). The system Xc-allows cysteine to enter the cell in its oxidized form and reduces it again to cysteine within the cell.

Glutathione peroxidase 4 (GPX4) is an oxidation inhibitor protein and belongs to the selenoproteins. GPX4 protects cells from lipid peroxidation by catalyzing the occurrence of reduction reactions, of which GSH is the most prevalent reducing agent (Ursini et al., 1995). GPX4 converts GSH to GSSG and also returns the phospholipid polyunsaturated fatty acid peroxides (PL-PUFA-OOH) to the corresponding phosphatidyl alcohol (PL-PUFA-OH) for reducing the accumulation of peroxidized lipids and inhibiting ferroptosis (Florez and Alborzinia, 2021). As a phospholipid hydroperoxide, Gpx4 is expressed or active under the regulation of selenium and GSH. Inhibition of the GPX4-GSH-cysteine axis is a significant factor contributing to ferroptosis.

## 3 Association between ferroptosis and COVID-19

### 3.1 Signitures of ferroptosis in COVID-19 multi-organ complications

**FIGURE 4:**
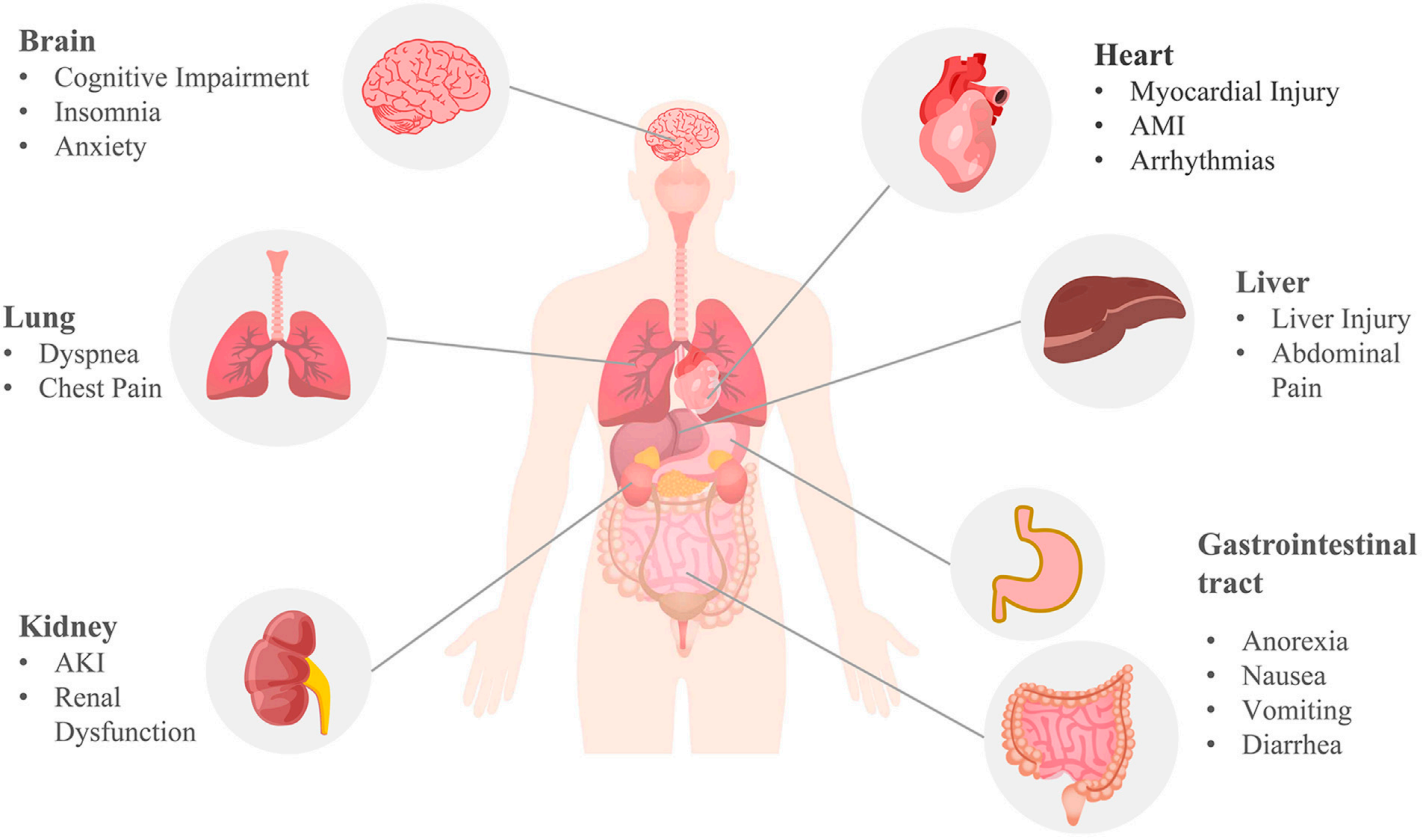
Multi-organ complications of COVID-19. COVID-19 involves the lungs, heart, brain, kidneys, liver, and gastrointestinal tract. Pulmonary symptoms include dyspnea and chest pain; common cardiovascular complications include myocardial injury, acute myocardial infarction (AMI), and arrhythmia; brain complications often include cognitive impairment, insomnia, anxiety, and other central nervous symptoms; acute kidney injury (AKI) and renal dysfunction are the main renal manifestations; anorexia, nausea, vomiting, diarrhea, abdominal pain, and liver injury are common digestive symptoms of COVID-19. The mechanism of COVID-19 multi-organ complications may be correlated with ferroptosis.

Although respiratory symptoms are the most common symptom of COVID-19, severe patients may experience pulmonary, cardiac, nephritic, neurological, gastric, and hepatic damage, as well as impaired immune and coagulation function (Gupta et al., 2020) (Figure 4). Moreover, some patients with long COVID may suffer from persistent dyspnea, chest pain, headache, and decreased mental status even after negative SARS-CoV-2 RNA tests or weeks to months following initiation of the disease. These symptoms appear to be common in heavy patients and groups of young people or children who do not require respiratory support, affecting the patient’s daily routine and survival after infection.

Angiotensin-converting enzyme 2 (ACE2) has been reported to be a common viral receptor (Perico et al., 2020). During infection, SARS-CoV-2 forms a complex by binding the tegument-expressed stinging protein (protein S) to the viral receptor ACE2, after which the complex is endocytosed, and the virus enters the host cell. The virus binds to ACE2 present in lung cells to infect the organism, resulting in symptoms including mild upper respiratory symptoms or severe dyspnea. The lungs of dead patients due to COVID-19 can be seen: severe endothelial damage, intracellular SARS-CoV-2, and extensive microangiopathy with vascular thrombosis (Ackermann et al., 2020). ACE2, a crucial enzyme of the renin-angiotensin-aldosterone system (RAAS), is expressed in multiple organs all over the system and is vital in sustaining blood pressure and cardiovascular, renal, immune, and nervous system homeostasis. Considering the association of SARS-CoV-2 and ACE2, Patel et al. (2017) have hypothesized that COVID-19 can cause an imbalance in RAAS. The broad distribution of ACE2 throughout the body may explain the extra-pulmonary manifestations of COVID-19, and it supports the idea that SARS-CoV-2 infection is a multi-system disease affecting the lungs. However, the pathogenesis of long COVID is not yet conclusive. The most supported theory is that it occurs through an autoimmune process with exaggerated innate immune responses and cytokine activation (Lechner-Scott et al., 2021). Ferroptosis plays an important role in the immune response and cytokine activation induced by SARS-CoV-2. In the late stages of infection, various cell deaths, including ferroptosis, promote inflammatory cytokine release, exacerbating immune and inflammatory system dysfunction and causing associated damage that ultimately leads to COVID-19 multi-organ complications (Sun et al., 2022). We describe the frequent organ complications in COVID-19 and ferroptotic signitures below.

#### 3.1.1 Heart

Data indicate that the heart is the second most affected organ with the entry of SARS-CoV-2 into the host through binding to ACE2 (Zou et al., 2020). Commonly reported cardiovascular complications involving COVID-19 include myocardial injury, acute myocardial infarction (AMI), and arrhythmias. Severe patients are usually accompanied by increased cardiovascular complications, including thoracic pain, tachypnea, fainting, tachycardia, and other common symptoms (Zou et al., 2020). Infection with the virus in the coronary region could trigger thrombosis and lead to acute coronary syndrome (Chieffo et al., 2020). Moreover, patients with COVID-19 who have underlying cardiovascular disease have been proven to have a higher chance of poor prognosis (Guo et al., 2020; Ni et al., 2020). However, given the limited evidence of direct cardiac infection by SARS-CoV-2 in animal models and patient autopsy samples, Nishiga et al. (2022) suggested that cardiac injury in COVID-19 could also be an indirect result of the cytokine storm. For example, ferroptosis during acute SARS-CoV-2 infection triggers the death of sinus node pacing cells, causing irreversible sinus node damage and ultimately leading to bradycardia (Han et al., 2022). Activation of the ferroptosis signaling pathway by viral infection may explain part of the mechanism of COVID-19 cardiovascular complications.

#### 3.1.2 Brain

Clinical data reveal that central nervous system symptoms are present in 36% of patients with COVID-19 (Mao et al., 2020). Inflammation and hypoxia in the brain caused by SARS-CoV-2 infection can impact the center nerve system, leading to various neuropsychiatric symptoms such as cognitive impairment, insomnia, and anxiety that can last for months after the respiratory symptoms have subsided (Boldrini et al., 2021). ACE2 is more abundant in the brainstem than in other brain regions. The viral proteins of SARS-CoV-2 and the evidence of pathological immunity were discovered in dead patients’ brainstems (Matschke et al., 2020). Furthermore, ferroptosis is connected with the pathology of several neuronal degeneration diseases, such as the Alzheimer as well as the Parkinson. It is hypothesized that the virus can damage the brain either through direct infection of the brain tissue or by inducing a series of pro-inflammatory and immune response pathways. Ferroptosis, associated with neuroinflammatory processes, may promote brain damage and psychiatric symptoms emerging in COVID-19 patients. Brain injury disorders, including cerebrovascular lesions, ischemic stroke, and cerebral hemorrhage, have been observed in COVID-19 patients. Furthermore, critically unwell patients have a high rate of brain injury than those who are not critically ill (Koralnik and Tyler, 2020; Lee et al., 2021). Therefore, severe SARS-CoV-2 infection has a strong potential to cause an acute stroke.

#### 3.1.3 Kidney

The kidney is one of the most frequently attacked targets of SARS-CoV-2. Acute kidney injury (AKI) is the most commonly observed renal impairment in clinical practice, and its pathogenesis is believed to be multifactorial. AKI could be brought on by the virus itself, hypoxia, or shock (Amann et al., 2021). Clinical studies have revealed that severe COVID-19 patients, even without a history of renal disease, can develop renal dysfunction or impairment (Ronco et al., 2020). Autopsy data from COVID-19 patients have indicated SARS-CoV-2 was present in various renal compartments, particularly in the parenchyma of the kidney, cells of glomerular epithelial, endothelial, and tubular (Puelles et al., 2020). An analysis conducted in the United Kindom has revealed that COVID-19 patients with chronic kidney disease had a higher mortality risk than those with other recognized risk factors, such as chronic cardiopulmonary disease (Williamson et al., 2020). Infecting SARS-CoV-2 could induce renal dysfunction or injury in patients without underlying renal disease and aggravate pre-existing renal damage, thereby increasing mortality risk. The virus can cause kidney injury through direct infection with ACE2, which is widely expressed in different kidney regions. Additionally, SARS-CoV-2 can aggravate kidney injury by inducing coagulation dysfunction or cytokine and complement activation. The kidney is vulnerable to oxidative stress, and excessive accumulation of ROS can cause kidney injury. Therefore, disruption of lipid metabolism is considered a common mechanism for the progression of various types of kidney diseases (Ratliff et al., 2016; Zhou et al., 2022). Inhibition of ferroptosis might have an improving effect on kidney injury induced by SARS-CoV-2 infection.

#### 3.1.4 Gastrointestinal tract

ACE2 is also expressed in the digestive system, including the duodenum, jejunum, and liver (Li et al., 2020). Therefore, the digestive system is susceptible to SARS-CoV-2 infection. Patients infected with SARS-CoV-2 frequently present with digestive symptoms in addition to the commonly reported respiratory symptoms. Anorexia, nausea, vomiting, diarrhea, abdominal pain, and liver injury are common digestive symptoms of COVID-19 and may appear during infection or after negative SARS-CoV-2 RNA tests (Chen et al., 2020; Brussow and Timmis, 2021). Diarrhea is the most frequently observed gastrointestinal symptom among COVID-19 patients. A study found viral RNA in stool samples from almost half of the patients, including those with negative respiratory tests (Cheung et al., 2020). SARS-CoV-2 infection can disrupt the adhesion and tight junctions between the endothelium and intestinal epithelium, leading to dysbiosis of the intestinal flora and immune activation (Guo et al., 2021). The virus causes damage to the gastrointestinal tract by activating innate immune cells and promoting the release of inflammatory factors, leading to a cytokine storm. After SARS-CoV-2 infects gastrointestinal cells via ACE2 may lead to RAAS dysregulation, exacerbating ionic imbalance and inflammation, affecting cellular metabolic status, flora composition, and cell viability, resulting in increasing gastrointestinal dysfunction in COVID-19 patients (Megyeri et al., 2021).

#### 3.1.5 Liver

The liver is an essential part of the digestive system with immune function and contains many cells associated with immune response. After the virus infects the liver, immune cells may become over-activated and secrete a large number of inflammatory factors, leading to cytokine storm and inducing ferroptosis, resulting in lung injury and ischemia, and hypoxia. Ischemia and hypoxia trigger systemic inflammatory response syndrome (SIRS), which can cause damage to vital organs throughout the body, including further liver cell damage and death (Tian and Ye, 2020). Therefore, ferroptosis may be one of the critical mechanisms of COVID-19 liver injury. It is worth noting that the expression of ACE2 in hepatocytes increases under liver fibrosis/cirrhosis conditions (Paizis et al., 2005). Patients with underlying liver disease and COVID-19 are more likely to suffer from viral attacks on their liver and are at a greater risk of developing severe COVID-19.

### 3.2 Link between SARS-CoV-2 infection and ferroptosis signaling pathway

**FIGURE 5:**
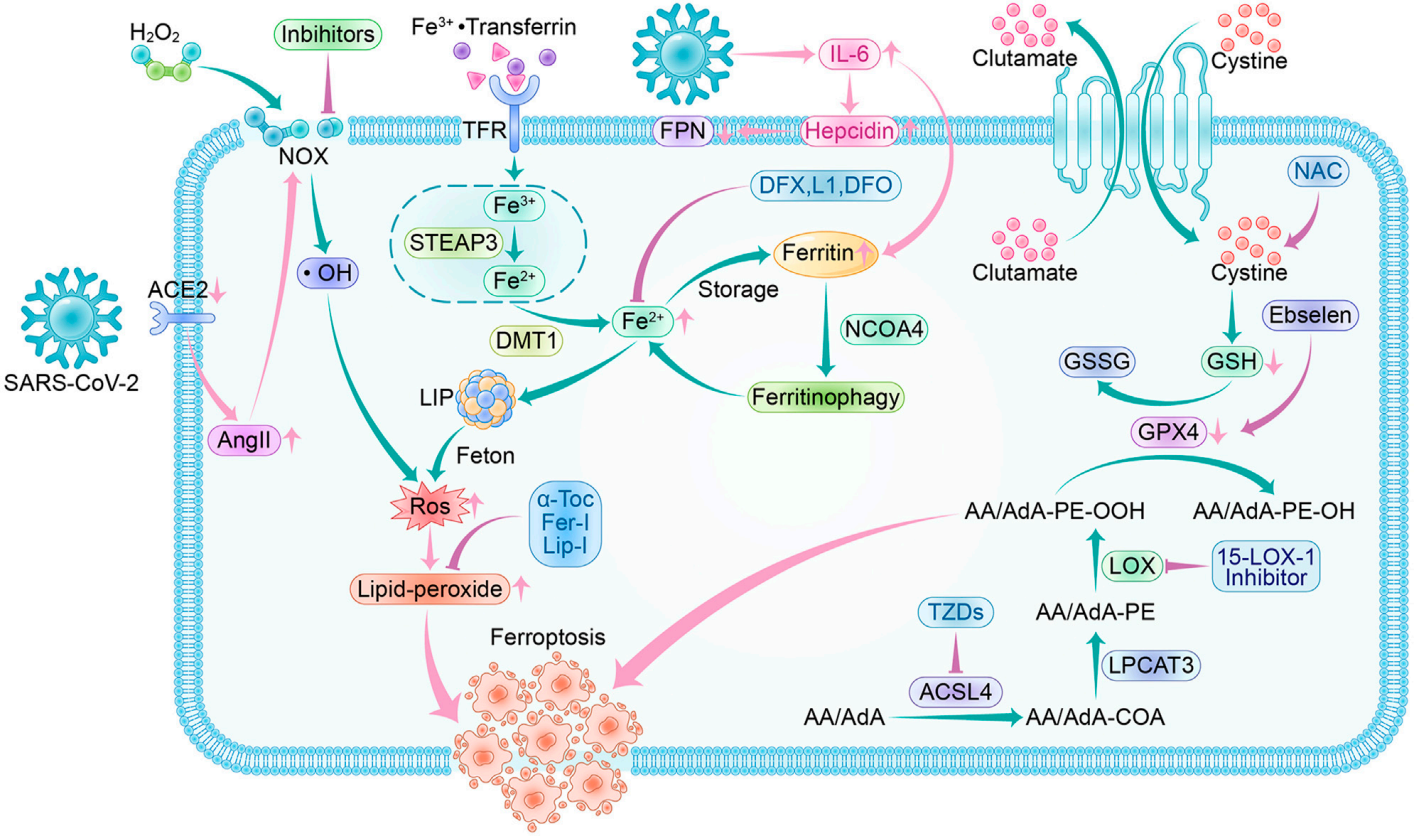
Ferroptosis pathway and inhibitors in SARS-CoV-2 infection. Crossover of SARS-CoV-2 infection and ferroptosis pathway:SARS-CoV-2 invades cells by binding to ACE2, and a feedback increase in circulating AngII triggers NOX activation leading to increased ROS production. During the acute phase of SARS-CoV-2 infection, a surge in IL-6 promotes hepcidin and ferritin synthesis. Hepcidin decreases FPN expression levels, resulting in a decrease in intracellular free iron output. The increased ferritin guarantees intracellular iron storage, and in inflammatory conditions, ferritin is degraded to release free iron, leading to a raised LIP, which generates ROS through the Fenton reaction. SARS-CoV-2 infection depletes intracellular GSH and attenuates Gpx4 activity, exacerbating lipid peroxidation. Ferroptosis inhibitors: NOX inhibitor, 15-LOX-1 inhibitor, and ACSL4 inhibitor-thiazolidinediones (TZDs) are the main oxidase inhibitors; α-Tocopherol (α-Toc), ferroportin-1 (Fer-1) and Liproxstatin-1 (Lip-1) can trap peroxyl radicals; they can prevent lipid peroxidation from occurring. Desferrioxamine (DFO), deferiprone (L1), and deferasirox (DFX) can form complexes with iron to reduce LIP, thus interrupting lipid peroxidation caused by iron overload. Ebselen acts as a selenium supplement to maintain GPX activity and N-acetylcysteine (NAC) increases GSH levels *in vivo*; they enhance the XC--GSH-Gpx4 axis to reduce lipid peroxide accumulation to resist ferroptosis.

Most patients who have acute COVID-19 exhibit significantly elevated serum levels of pro-inflammatory cytokines. This can cause a cytokine storm, leading to an abnormal systemic inflammatory response. Once the cytokine storm occurs, the immune system response becomes uncontrollable and can attack multiple tissues and organs of the body, causing multi-organ damage (Xu Z. et al., 2020). COVID-19 patients also experience systemic hyperinflammation, characterized by elevated ROS and cytokine storm (Girelli et al., 2021). As a result, there may be a link between SARS-CoV-2 infection and the ferroptosis signaling pathway. Using ferroptosis inhibitors to block the signaling pathway may reduce the multi-organ damage caused by SARS-CoV-2 (Figure 5).

#### 3.2.1 Lipid peroxidation and coagulation in SARS-CoV-2 infection

It was demonstrated that angiotensin II (Ang II), hyperglycemia, hyperlipidemia, or hypoxia are associated with NOX activation, leading to excessive production of mitochondrial ROS (Dikalov and Nazarewicz, 2013). SARS-CoV-2 invades cells by binding to ACE2, which leads to downregulation of ACE2 and feedback increased Ang II in the circulation, thus triggering NOX activation, resulting in oxidative stress and inflammation (Hikmet et al., 2020). Clinical observations indicate that thrombotic complications have become an essential issue in patients with COVID-19 (Guan et al., 2020). It has been reported that NOX2 was triggered in COVID-19, with higher values in critically ill patients than in non-critically ill patients, strongly linked to thrombotic events (Violi et al., 2020).

Inflammation initiates coagulation through tissue factor TF, which is present in monocytes and vascular endothelial cells (Iba et al., 2019). Oxidative stress products have been shown to promote TF expression and initiate monocyte inflammatory programs. At the same time, activated endothelial cells are fully responsible for initiating coagulation using TF-expressing inflammatory monocytes (Owens et al., 2012; von Bruhl et al., 2012). The accumulation of lipid peroxidation products in the COVID-19 patients’ lungs and cardiovascular system may also be involved in the process of coagulation initiation (Merad and Martin, 2020), contributing to a hypercoagulable state of blood in patients. A clinical study supported the association of COVID-19 severity with a hypercoagulable state (Helms et al., 2020). The dislodgement of the formed thrombus caused by the hypercoagulable state leads to fatal complications such as strokes in patients. In addition, iron oxide accelerates serum coagulation by interacting with coagulation cascade proteins. Methemoglobinemia and free iron may also contribute to the COVID-19 hypercoagulable state (Jankun et al., 2014). Lipid peroxidation and iron overload are ferroptosis-related factors, so we speculate that the increased incidence of cardiovascular and cerebrovascular complications in patients with moderate to severe COVID-19 may be related to iron death.

#### 3.2.2 Iron metabolism in SARS-CoV-2 infection

It has been shown in clinical studies that COVID-19 patients have abnormal iron metabolism, with patients having low serum iron levels but elevated serum iron levels after treatment (Bellmann-Weiler et al., 2020; Zhao et al., 2020). Besides, those COVID-19 patients with significantly lower serum iron concentrations and transferrin levels are commonly anemic, indicating a potential inverse relationship between serum iron levels and the severity of COVID-19. Hepcidin, an antimicrobial cysteine-rich peptide synthesized and secreted by the liver, is abundantly expressed during the immune process and plays a role in regulating iron homeostasis in the body. It could regulate the expression levels of iron transport-related proteins, especially FPN, to decrease iron export from cells and increase iron storage. During the emergency phase of SARS-CoV-2 infection, the abrupt increase of IL-6 in a highly inflammatory state promotes the synthesis of hepcidin and ferritin.

Additionally, pathogens’ iron may be deprived and coupled with transferrin to enter cells via TFR1, increasing the amount of intracellular iron. Iron can be sequestered in cells by hepcidin, leading to decreased iron efflux from cells, while increased ferritin allows sufficient iron stores. Notably, it has been suggested that ferritin leaks out of cells damaged by inflammation, having lost most of its iron in the process, and that iron remains in the cell unconnected (Kell, 2010). A rise in intracellular free iron caused by the above pathways may explain the decrease in serum iron levels in patients with COVID-19. It may trigger further cellular damage, which ultimately results in ferroptosis. In severe COVID-19, as a consequence of cell death and tissue damage, intracellular ferritin is released out of the cells, with excess ferritin accumulating in the body to form hyperferritinemia (Kell and Pretorius, 2014). Therefore, elevated serum ferritin levels are typically linked to systemic inflammation. Methemoglobinemia is largely considered to be an indicator of severe COVID-19 correlation. In hospitalized COVID-19 patients, the risk of death increases roughly nine-fold when blood ferritin levels exceed 300 μg/L (Goldberg et al., 2020).

#### 3.2.3 Antioxidant system in SARS-CoV-2 infection

Endogenous GSH deficiency may be closely related to factors such as age, gender, and concurrent chronic. Viral replication accelerates cysteine depletion, and COVID-19 patients are vulnerable to endogenous GSH depletion which has been demonstrated to function as a critical player in specific viral infections (Polonikov, 2020). A study showed that a decrease in GSH was linked to more ROS and complications, so patients with increased GSH levels had reduced ROS production and faster recovery (Horowitz et al., 2020). As an important antioxidant and free radical scavenger in the body, GSH is synthesized in the liver and functions in hepatic biochemical metabolism. One of the key contributors to liver damage in COVID-19 may be the formation of lipid peroxides brought on by GSH shortage, which in turn accelerates GSH depletion and exacerbates endogenous GSH insufficiency. Besides, immune responses induced by SARS-CoV-2 infection may also contribute to liver damage as one of the common factors (Bangash et al., 2020).

An underlying study infected African green monkey kidney (Vero) cells with SARS-CoV-2 and found that SARS-CoV-2 resulted in a significant reduction in mRNA expression of endoplasmic reticulum-resident selenoproteins, which is strongly correlated with selenium by GPX4 expression or activity, so infection with SARS-CoV-2 may be related to suppression of GPX4 (Wang et al., 2021). Available data suggest that infecting with SARS-CoV-2 would induce low levels of GSH and decreased expression of GPX4 protein, both of which are essential aspects of ferroptosis (Dixon et al., 2012). Ferroptosis may explain the clinical phenomenon that severe COVID-19 patients without a background of renal disease nevertheless develop renal dysfunction or impairment (Cheng et al., 2020). Notably, GPX4-deficient T cells undergo rapid accumulation of lipid peroxide (Matsushita et al., 2015). Eventually, the T cells go into ferroptosis due to the lack of GPX4, resulting in a weakened body defense system unable to effectively defend against viral infections, which may be one of the crucial reasons for COVID-19 progressing to severity.

## 4 COVID-19 potential treatment based on ferroptosis

Using ferroptosis inhibitors to block the signaling pathway may reduce multi-organ damage from SARS-CoV-2 infection. Both enzymatic and non-enzymatic processes in mammalian cells can generate ROS, leading to lipid peroxidation. The two primary categories of methods to block this process are oxidase inhibitors and lipid autoxidation inhibitors. Free iron is a crucial factor in the induction of ROS production, and controlling its levels *in vivo* is critical for preventing ferroptosis. Iron chelating agents and hepcidin inhibitors are two effective methods for reducing free iron levels. Additionally, maintaining appropriate levels of GSH and activity of GPX4 expression is crucial for the GPX4-GSH-cysteine axis to function effectively against ferroptosis. Some treatments for viral infections have also been found to inhibit ferroptosis, potentially reducing complications of COVID-19.

### 4.1 Inhibitors of lipid peroxidation

#### 4.1.1 Oxidase inhibitors

In lipid peroxidation with the involvement of oxidases, oxidase inhibitors can reduce the production of ROS and inhibit ferroptosis. 15-LOX-1 inhibitors may block ferroptosis and have positive performance in treating ischemic and hemorrhagic stroke (Yigitkanli et al., 2013; Wenzel et al., 2017). This may offer an alternative direction for improving brain injury in COVID-19 patients. Currently, a variety of NOX inhibitors with new structures are emerging. However, due to the short research period, some NOX inhibitors are still in the preliminary research stage, except for a few inhibitors under clinical trials. Recent studies, however, have revealed that AA metabolites of the LOX pathway have a signal link with NOX and can activate NOX-mediated ROS production in various cells (Cho et al., 2011). Consequently, we turned to the LOX pathway of AA and found a complex tissue-specific interaction between LOX and GPX4 (Brutsch et al., 2016). LOX has many isoforms. It has been reported that inhibition of multiple lipoxygenases is more protective for cells than targeted inhibition of a single lipoxygenase (Yang et al., 2016). Regrettably, more studies are necessary to validate how oxidase inhibitors impact ferroptosis.

As mentioned previously, ACSL4 is essential for the oxidation of AA by LOX. Therefore, the inhibition of ferroptosis could be achieved if ACSL4 is inhibited from interrupting the process of lipid peroxidation. Studies have shown significant resistance to ferroptosis for ACSL4-deficient cells even when the GPX4 gene was inactivated (Doll et al., 2017), adding to the conviction that ACSL4 may act as a target for ferroptosis inhibition. The peroxisome proliferator-activated receptor γ (PPARg) agonist, thiazolidinediones (TZDs), selectively inhibits ACSL4 (Kim et al., 2001). TZDs like rosiglitazone (Rosi), pioglitazone (PIO), and troglitazone (Tro), show significant inhibition in a model of ferroptosis cells induced by the ferroptosis inducer RSL-3. Both ACSL4-deficient and Acsl4-non-deficient cells treated with Rosi demonstrated a decrease in AA/AdA-PE (Doll et al., 2017). By inhibiting ACSL4, TZDs are believed to reduce the availability of substrates and prevent lipid peroxidation. It has been reported that the ACSL4 inhibitor Rosi mitigated pathological kidney and lung injury based on inhibiting cellular ferroptosis (Xu Y. et al., 2020; Wang et al., 2022), which may give hope for the treatment of COVID-19 complicated by multi-organ injury. Moreover, TZDs have been developed as an insulin sensitizer and may be optional for COVID-19 patients with underlying diabetes. More clinical trials are expected to validate the safety and efficacy of TZDs for ferroptosis inhibition.

#### 4.1.2 Lipid autoxidation inhibitors

In non-enzymatic lipid peroxidation, lipid autoxidation inhibitors represented by RTAs protect lipids from autoxidation. RTAs are compounds that can interact with chain-carrying radicals. They are also referred to as chain-breaking antioxidants. α-Tocopherol (α-Toc), the most biologically active form of vitamin E, is a characterization of the activity in RTAs (Burton and Ingold, 2002). α-Toc is a typical antioxidant that traps peroxyl radicals and exceeds the ability of other lipid substrates to be oxidized by peroxyl radicals. Interestingly, vitamin E has recently been reported to have another possible function - direct inhibition of lipoxygenases, which may be achieved by competing for the substrate binding site with lipoxygenases (Kagan et al., 2017). As COVID-19 therapeutic agents, vitamin E supplements can reduce the damage caused by ferroptosis with several organs, including the lungs, kidneys, liver, intestines, heart, and nervous system. Furthermore, phenprocoumon, a vitamin K antagonist, was shown to significantly exacerbate ferroptotic cell death *in vitro* and significantly worsen the course of AKI in mice (Kolbrink et al., 2022). Vitamin K, as an antioxidant, can also prevent lipid peroxidation and thus inhibit ferroptosis, which has a therapeutic effect in COVID-19 patients with AKI.

In addition, ferroportin-1 (Fer-1) and Liproxstatin-1 (Lip-1) have been recognized in recent years as RTAs that effectively inhibit ferroptosis because of their ability to trap peroxyl radicals with acyl chains in lipid bilayers, having a strong activity to slow down the accumulation of lipid peroxides (Zilka et al., 2017). Fer-1 and Lip-1 are more potent in terminating lipid peroxidation compared to α-Toc. It may attribute to the fact that they are subsequently converted to nitrogen oxides, which are good RTAs (Haidasz et al., 2016). In the works of Krainz et al. (2016), Fer-1 and Lip-1 could inhibit ferroptosis in an *in vitro* cellular system. Lip-1 was the first Liproxstatin-like molecule to be identified, and it showed good pharmacological properties in the low nanomolar range with a short plasma half-life. In animal experiments, Lip-1 has been shown to effectively reduce liver and kidney-related disorders such as fatty liver and renal fibrosis by inhibiting ferroptosis (Zhang et al., 2021; Tong et al., 2023). Importantly, such molecule was shown to counteract acute renal failure in a Gpx4-deficient model and to have ferroptosis-inhibiting activity *in vivo* (Friedmann Angeli et al., 2014). Fer-1 eliminates lipid hydroperoxides in the presence of iron reduction. Fer-1 might interact with iron, as same as other antioxidants or complexing molecules, forming complexes to reduce lipid peroxidation formation. It has demonstrated that Fer-1 can effectively inhibit the oxidation of cell membranes and possess a notable protective effect on AKI (Skouta et al., 2014). Further studies are necessary to support the hypothesis that such inhibitors can reduce the complications of COVID-19. Although Fer-1 and Lip-1 are generally considered safe, there are limited studies on their potential adverse reactions. Therefore, it is imperative to exercise caution in the dose control of future *in vivo* trials.

### 4.2 Iron depletion methods

Free iron can catalyze the production of ROS in Fenton reaction, and excessive production of ROS in the presence of iron overload can lead to oxidative stress and damage DNA, lipids, and proteins (Evans et al., 2004). Therefore, excess iron can lead to cellular damage, which is harmful to the organism. The SARS-CoV-2 requires iron to replicate and function. As described previously in 3.3.2, LIP increases during the acute phase of infection by a mechanism involving the deprivation of additional iron from the pathogen. Currently, the treatment for iron overload is usually iron chelation therapy. This treatment uses iron complexing agents to compete with the body’s natural chelator transferrin for iron and reduce LIP to interrupt the iron-catalyzed lipid peroxidation process. Desferrioxamine (DFO), deferiprone (L1), and deferasirox (DFX) are the three types of iron chelators most frequently used in the globe.

DFO is a clinically used iron chelator derived from *streptomyces* polymyxa and is a hexadentate complex that forms a 1:1 Fe^3+^/DFO complex at physiological PH. DFO is generally administered intravenously for a prolonged period, requiring five to 7 days of infusion a week. For this reason, oral iron chelators have become a common choice (Franchini and Veneri, 2004). L1, an FDA-approved oral iron chelator, is a bidentate chelator that forms 3L1-1Fe complexes with iron and is comparable in efficacy to DFO. L1 is rapidly absorbed after oral administration, usually peaking 45 min after ingestion, and the widely adopted recommendation is to take three doses of L1 daily (Banerjee et al., 2019). Notably in COVID-9 patients, L1 can restore T cell resistance to the virus infection by increasing the expression of IFN-R2 on the surface of activated T cells (Regis et al., 2005; Perricone et al., 2020). DFX is also a commonly used oral iron chelator, a tridentate complex that can form 2DFX-1Fe complexes with iron, and its efficacy is also comparable to that of DFO. DFX reaches peak blood levels within one and a half to 4 h after oral administration, with a half-life ranging from 8 to 16 h, and is generally administered once daily (Stumpf, 2007). DFX and DFO have been reported to reduce tissue fibrosis by inhibiting free radical production and tissue infiltration of macrophages and significantly reducing IL-6 levels (Darwish et al., 2015). Therefore, DFX and DFO can treat COVID-19 patients with tissue damage caused by increased LIP and subsequent liver injury complicated by fibrosis. The mechanism of action differs between iron chelators, with DFO exerting a direct effect by inducing autophagy to promote ferritin degradation in the lysosome, while L1 and DFX may chelate intracellular free iron and take iron from ferritin before proteases break it down (Temraz et al., 2014).

Yet, iron complexation toxicity is a major problem, especially in patients with inadequate iron stores (Kolnagou et al., 2014). DFX is not recommended for iron-loaded patients with a serum ferritin below 500 μg/L. Patients receiving DFX are regularly monitored for renal function, with discontinuation of the drug recommended for patients with persistently elevated serum creatinine levels. DFO is relatively safe and has a much lower incidence of serious toxicities, but restrictions on the use of DFX still apply in patients with low iron stores. It is noted that ocular and auditory toxicity has also been reported with DFO when used for ophthalmic disease (Orton et al., 1985). The most serious reported toxicities of L1 are reversible granulocyte deficiency and neutropenia, for which blood counts are recommended every one to 2 weeks to prevent L1 toxicity. Besides, less severe toxic effects of L1 include gastric intolerance, joint pain, and zinc deficiency (Kolnagou et al., 2014). Notably, hepcidin plays a vital function, including iron regulation in SARS-CoV-2 infection as described earlier in 3.3.2. Studies have shown that the hepcidin inhibitor dalteparin improves symptoms in diabetic COVID-19 patients by properly regulating and reducing oxidative stress and inflammation (Zeinivand et al., 2022). This finding is beneficial for the prospect of developing dalteparin as a therapeutic agent for COVID-19 patients with the underlying diabetic disease.

### 4.3 GPX4-GSH-cysteine axis protector

SARS-CoV-2 infection disrupts the balance between the body’s oxidative and antioxidant systems, preventing the timely clearance of ROS. As mentioned above, the GPX4-GSH-cysteine axis is one of the most critical antioxidant systems against ferroptosis, and selenium has a protective effect on GPX4 activity. However, COVID-19 patients with systemic inflammation have decreased hepatic selenase production, which lowers intraplasma selenium (Heller et al., 2021). Thus COVID-19 patients have GPX4 inhibition due to selenium deficiency, leading to ferroptosis. A related study showed that Ebselen was suggested as a low cytotoxic organoselenium compound for the clinical treatment of COVID-19 (Banerjee et al., 2021), as it could reduce the virus replication in the experiment. Furthermore, Ebselen can be used as a selenium supplement to maintain Gpx activity to enhance the antioxidant system. SARS-CoV-2 infection may lead to GSH deficiency, and patients with severe COVID-19 are often accompanied by liver damage by a mechanism related to ferroptosis. Maintaining GSH levels not only resists ferroptosis but also has great significance in protecting liver function itself. Since cysteine is a precursor of GSH synthesis, maintaining adequate cellular cysteine levels can hinder GSH depletion and enable GPX4 to scavenge lipid peroxidation products continuously (Stockwell et al., 2017). N-acetylcysteine (NAC) is a cysteine prodrug used to treat acetaminophen-induced liver failure, critically ill patients with sepsis, and mucus loss in chronic obstructive pulmonary disease. On the one hand, NAC can act as a precursor of GSH and exert an antiferroptosis effect by enhancing the Xc--glutathione--GPX4 axis. On the other hand, it has been shown that NAC can act as a free radical scavenger to counteract IL-6-induced ferroptosis in bronchial epithelial cells while possibly alleviating respiratory symptoms in COVID-19 patients (Han et al., 2021). Recently, there has been evidence that NAC is available for preventing and treating COVID-19 as an adjuvant drug (Shi and Puyo, 2020).

### 4.4 Others

Lactoferrin (LF) is a first-line defense protein that has a major role in the maturation and regulation of immune system function. Some studies suggested that LF enhances immunity against SARS–CoV–2 by enhancing intracellular antiviral mechanisms and could be a potential adjuvant therapy for COVID–19 (AlKhazindar and Elnagdy, 2020; Chang et al., 2020). In addition, LF, as an iron-binding protein, possesses the ability to sequester free iron and prevent injury-induced oxidative stress that may be associated with ferroptosis, which can eventually lead to severe tissue necrosis. LF can be used for prophylaxis or as a therapeutic agent administered by multiple routes (including oral administration) to individuals at risk of SARS-CoV-2 infection, especially those with impaired innate immune function (Zimecki et al., 2021). Hypoxia-inducible factor (HIF), the effective substrate of HIF prolyl hydroxylase (HIF-PHD), similarly activates several genes involved in glucose metabolism, intracellular acidity regulation, vasculature, iron overload, mitosis, and other physiological processes (Wang and Semenza, 1995). Inhibitors of HIF-PHD can promote endogenous erythropoietin (EPO) by stabilizing and activating HIF and erythropoiesis (Joharapurkar et al., 2018). Since EPO treatment has anti-inflammatory and healing characteristics, people with moderate to severe COVID-19 are likely to benefit from it. Poloznikov et al. (2020) suggested that HIF-PHD inhibitors can counteract ferroptosis through various interactions with iron but also have the potential to cause greater damage. HIF-PHD inhibitors are a prospective therapy for COVID-19 adverse effects since they can inhibit ferroptosis as well as the entry of the virus into cells. Moreover, Coenzyme Q (CoQ), an essential antioxidant and anti-inflammatory compound in the body, is regarded as a lipophilic antioxidant that traps free radicals. As a CoQ reducer, ferroptosis suppressor protein 1 (FSP1) regenerates CoQ in the plasma membrane using NADPH. The Xc—glutathione—GPX4 axis is assumed to be a parallel system to the FSP1-CoQ10-NADPH pathway, and the two work together to combat ferroptosis and lipid peroxidation (Doll et al., 2019).

## 5 Discussion

COVID-19 is still spreading worldwide, negatively impacting human health and life. Severe patients are at high risk of complicated multi-organ failure and immune and coagulation dysfunction. Even mildly ill patients may still have persistent respiratory, cardiovascular, neurological, and digestive sequelae. It may be attributed to the wide distribution of ACE2 throughout the body, and SARS-CoV-2 damages multiple tissues and organs through ACE2. Besides, COVID-19 causes high systemic inflammation characterized by elevated ROS and cytokine storm (Girelli et al., 2021). The imbalance between oxidative and antioxidant systems triggered by SARS-CoV-2 infection causes elevated ROS, which exacerbates the corresponding acute/chronic inflammatory process, ultimately leading to multi-organ damage in the body. COVID-19 primarily affects multiple organs, including the lungs, heart, brain, liver, and gastrointestinal tract. Fatigue, dyspnea, cardiac arrhythmia, anxiety, insomnia, abdominal pain, diarrhea, and vomiting are among the symptoms. We are eager to discover new ways to reduce the complications of COVID-19, thereby easing the burden of life and gradually restoring social order as soon as possible. During our research, we discovered that ferroptosis could be a significant breakthrough.

Ferroptosis is a process that involves three primary metabolisms of thiols, lipids, and iron (Tang et al., 2021). It is a relatively passive process that results in cellular destruction or imbalance, mainly due to intracellular iron overload or disruption of normally active antioxidant mechanisms. This results in reactions of lipid peroxidation that depend on iron and the buildup of lipid peroxides. The GPX4-GSH-cysteine axis is the most crucial component of the antioxidant system that protects against ferroptosis. When GSH levels are low, this axis is inhibited, which indirectly inactivates GPX4. The inhibition of the antioxidant system leads cells can not clear the accumulated lipid peroxide, which compromises the cell membrane and ultimately results in ferroptosis.

There may be a crossover between the SARS-CoV-2 infection process and the ferroptosis pathway. Infection with SARS-CoV-2 triggered inflammation, and increased cytokines such as IL-6 promoted the synthesis of hepcidin and ferritin. While the high level of inflammation causes depletion of cells, resulting in the release of free iron from intracellular iron-storing ferritin, accompanied by accumulation of free iron in cells but the leakage of ferritin into the blood leads to hyperferritinemia. Iron is required for SARS-CoV-2 replication. The extracellular iron could be transported to the cell through the transporter protein, which eventually increases the intracellular LIP resulting in the active Fenton reaction and the production of lipid ROS. SARS-CoV-2 invades the cell by binding to ACE2, which leads to the downregulation of ACE2 and, therefore, the feedback increase of Ang II in circulation. Ang II triggers the activation of NOX, leading to oxidative stress. Clinical studies have shown that infection with SARS-CoV-2 leads to GSH deficiency and GPX4 inhibition in the body. Thus the antioxidant system is inhibited, and GSH is unable to reduce lipid peroxides in the presence of GPX4. In addition, oxidative stress promotes TF expression to initiate coagulation, and a hypercoagulable state, associated with thrombosis in patients with severe COVID-19, may be a marker of ferroptosis. Also, in the presence of cellular iron overload, hyperproteinemia in COVID-19 patients marks a worse prognosis and higher mortality in COVID-19 patients.

This paper discusses the possible therapeutic effects of lipid peroxidation inhibitors, iron complexing agents, and GPX4-GSH-cysteine axis protectors for patients based on the potential connection between COVID-19 complications and ferroptosis. Lipid peroxidation inhibitors are mainly divided into oxidase inhibitors and autoxidation inhibitors. As an ACSL4 inhibitor, TZDs not only interrupt the oxidation of AA or AdA to reduce the pathological damage caused by COVID-19 but also acts as an insulin sensitizer. Therefore, patients with severe COVID-19 with underlying diabetic disease may be considered to use hepcidin inhibitors or rosiglitazone to inhibit ferroptosis and mitigate the associated symptoms. Fer-1 and Lip-1 are currently commonly used ferroptosis inhibitors, and they have been shown to inhibit ferroptosis as RTAs. Regrettably, their effects on COVID-19 patients have not been clarified yet. The main treatment for iron overload is the use of iron chelators, which reduce ROS from the Fenton reaction by binding to free iron. Some studies have demonstrated the effect of iron chelators in reducing tissue fibrosis, which can prevent liver injury complicated by severe COVID-19 due to increased LIP. However, it has to be taken into account that iron levels in the patient should not be too low while using iron complexing agents. Hepcidin inhibitors can also treat iron overload, thereby reducing oxidative stress and inflammation. It has surprisingly been reported to improve the symptoms of COVID-19 in diabetic patients. GPX4-GSH-cysteine axis protectors include selenium supplements and NAC, which together enhance the anti-ferroptosis effect of the GPX4-GSH-cysteine axis. Selenium supplements enhance the activity of Gpx4, and NAC not only scavenges free radicals but also acts as a precursor of GSH. GPX4-GSH-cysteine axis protectors include selenium supplements and NAC, which together act as anti-ferroptosis agents. GSH levels and Gpx4 activity are maintained to counteract the tissue and organ damage caused by SARS-CoV-2 infection and to reduce the severity of COVID-19. Other potential treatments for COVID-19-based ferroptosis include LF and HIF-PHD inhibitors. LF effectively enhances immunity to SARS-CoV-2, and HIF-PHD inhibitors are helpful in blocking virus entry into cells, prospective treatments for severe COVID-19 complications.

In conclusion, there is an association between ferroptosis and the multi-organ complications of COVID-19. Although partial inhibition of ferroptosis has been recorded clinically as adjuvant therapy for COVID-19 with desirable results (Shi and Puyo, 2020; Banerjee et al., 2021; Zeinivand et al., 2022), the definitive mechanism of ferroptosis inhibitors for preventing multi-organ damage in COVID-19 has not been illuminated. More clinical studies on the effects of inhibiting ferroptosis on COVID-19 are expected to emerge providing a new method to reduce COVID-19 complications.
